# Clinical Innovation Poster Abstracts

**DOI:** 10.1080/24740527.2020.1765648

**Published:** 2020-05-15

## Tele-Advice in Chronic Pain Management by Nurse

Kari Blessing^a,*^, Krista Aktug^a^, Ruth Hanis^a^, Lori Montgomery http://orcid.org/0000-0001-6338-6455^b,c^, Jennifer Kirker^a^ and Maria Vinnik^a^

^a^Calgary Pain Program, Alberta Health Services, Calgary, Alberta, Canada; ^b^Department of Family Medicine, University of Calgary Calgary Canada; ^c^Anesthesiology, Perioperative and Pain Medicine, Calgary Alberta, Canada

**CONTACT** Kari Blessing kari.blessing@ahs.ca

© 2020 The Author(s). Published with license by Taylor & Francis Group, LLC.

This is an Open Access article distributed under the terms of the Creative Commons Attribution License (http://creativecommons.org/licenses/by/4.0/), which permits unrestricted use, distribution, and reproduction in any medium, provided the original work is properly cited.

The In-Hospital Chronic Pain Consult Service (CPCS) was established by the Calgary Chronic Pain Center (CPC) in 2007 to provide treatment recommendations and education to healthcare staff caring for adult patients experiencing chronic pain in the acute care environment. Since then, our service has evolved to become a fully autonomous Nurse Practitioner (NP) service.

The CPCS offers a unique model of care delivery that utilizes NPs to their full scope of practice, including prescribing of controlled drugs and substances, and delivery of expert care within an evolving chronic pain landscape amidst the opioid crisis.

The CPCS launched with Specialist LINK© in July 2017, an innovative service that connects community providers and specialists in the Calgary area. Specialist LINK© includes real-time, tele-advice line for physicians and NP’s, clinical care pathways, and other resources that help to improve communication and collaboration between community providers and specialty care. We will present evaluation data indicating improved access to chronic pain specialists and provider satisfaction with the advice received.

The CPCS has evolved into a distinctive model of care within chronic pain management in Calgary. The utilization of the NP role to provide tele-advice for chronic pain management is a unique model of care delivery that can serve as a foundation for improving care for individuals and their families experiencing chronic pain.

## The Evolution of the Calgary In-Hospital Chronic Pain Consult Service

Ruth Hanis^a,*^, Krista Aktug^a^, Kari Blessing^a^, Lori Montgomery http://orcid.org/0000-0001-6338-6455^b,c^, Jennifer Kirker^a^ and Maria Vinnik^a^

^a^Calgary Pain Program, Alberta Health Services, Calgary, Alberta, Canada; ^b^Department of Family Medicine, University of Calgary, Calgary, Canada; ^c^Anesthesiology, Perioperative and Pain Medicine, University of Calgary, Calgary, Alberta, Canada

**CONTACT** Ruth Hanis ruth.hanis@ahs.ca

© 2020 Alberta Health Services. Published with license by Taylor & Francis Group, LLC.

This is an Open Access article distributed under the terms of the Creative Commons Attribution License (http://creativecommons.org/licenses/by/4.0/), which permits unrestricted use, distribution, and reproduction in any medium, provided the original work is properly cited.

Initially established by the Calgary Chronic Pain Center (CPC) in 2007, the In-Hospital Chronic Pain Consult Service (CPCS) was developed to provide treatment recommendations and education to healthcare staff caring for adult patients experiencing chronic pain in the acute care environment. Since then, our service has evolved to become a fully autonomous Nurse Practitioner (NP) service.

Various developments have contributed to the CPCS becoming a unique model of care delivery in the acute care setting. These include utilization of NPs to their full scope of practice, including prescribing of controlled drugs and substances; the need for expert knowledge and experience in the evolving chronic pain landscape amidst the opioid crisis; and the support of physicians at the CPC.

The CPCS has progressed to providing treatment recommendations in the four acute care sites, extending into the community through discharge recommendations and via a tele-advice line. We also offer educational opportunities for healthcare professionals, provide mentorship and a supportive learning environment for Clinical Clerks and Residents, and work as part of an interdisciplinary team in the NP clinic at the CPC.

The CPCS has evolved into a distinctive model of care within chronic pain management in Calgary. This model of care can serve as a foundation for expert-care delivery in the complex area of chronic pain management across Canada.

## Implementation of a Rapid Access Clinic for Paediatric Complex Regional Pain Syndrome: A Quality Improvement Project

Giulia Mesaroli^a,*^, Geraldine Cullen-Dean^b^, Catherine Munns^c^ and Stephen Brown^b^

^a^Department of Physical Therapy, The Hospital for Sick Children, University of Toronto, Toronto, Ontario, Canada; ^b^Department of Anesthesia and Pain Medicine, The Hospital for Sick Children, Toronto, Ontario, Canada; ^c^Department of Anesthesia and Pain Medicine, Department of Psychology, The Hospital for Sick Children, Toronto, Ontario, Canada

**CONTACT** Giulia Mesaroli giulia.mesaroli@sickkids.ca

© 2020 The Author(s). Published with license by Taylor & Francis Group, LLC.

This is an Open Access article distributed under the terms of the Creative Commons Attribution License (http://creativecommons.org/licenses/by/4.0/), which permits unrestricted use, distribution, and reproduction in any medium, provided the original work is properly cited.

**Background**: Complex regional pain syndrome type 1 (CRPS-1) is a painful condition of a limb characterized by a constellation of symptoms that occur most typically after a minor trauma. In pediatrics, the average age of onset is 12 years, is more prevalent in females, and most commonly occurs in the lower limb. Clinical expertise suggests that early access to intervention and treatment may lead to a more favorable prognosis. The International Association for the Study of Pain suggests a 1-week wait-time for acute onset CRPS-1 to receive pain specialist assessment. The Chronic Pain Clinic at The Hospital for Sick Children in Toronto, Canada, implemented a Rapid Access Clinic (RAC) for pediatric CRPS-1 as a quality improvement project. Main outcomes included (1) reduction in wait-times for interprofessional assessment; (2) reduction in wait-times for physical and psychological therapy; (3) feasibility and (4) acceptability.

**Methods**: The RAC was implemented in April 2019. Patients referred to our center with suspected CRPS-1 underwent a rapid triage process, interprofessional assessment (including an Anesthesiologist, Clinical Nurse Specialist, Physiotherapist and Psychologist), and rapid access to treatments.

**Results**: Between April 2019 - March 2020, 24 patients were referred to the RAC. Wait-times were significantly reduced for triage (mean = 4.3 days), assessment (mean = 12.5 days) and intervention (mean = 9.5 days physiotherapy, mean = 13.6 days psychology). Data obtained from patient and family satisfaction surveys and staff focus group yielded highly positive results.

**Discussion**: This novel approach to assessing pediatric CRPS-1 is feasible, acceptable and resulted in a significant reduction in wait-times. Further evaluation is needed to understand if early intervention lead to improve outcomes in this population.

## Validity of Somatic Symptom Disorder in a Sample of Patients with Chronic Pain

Gregory Tippin^a,*^ and Laura Katz^a^

^a^Michael G. DeGroote Pain Clinic, McMaster University Medical Centre, Hamilton, Ontario, Canada

**CONTACT** Gregory Tippin tipping@hhsc.ca

© 2020 The Author(s). Published with license by Taylor & Francis Group, LLC.

This is an Open Access article distributed under the terms of the Creative Commons Attribution License (http://creativecommons.org/licenses/by/4.0/), which permits unrestricted use, distribution, and reproduction in any medium, provided the original work is properly cited.

**Introduction**: Somatic Symptom Disorder (SSD) was introduced in DSM-5 as a replacement for DSM-IV Pain Disorder, where SSD includes a specifier for patients with predominantly pain. To date, SSD has not been widely examined within patients with chronic pain, despite the importance of this diagnosis within chronic pain populations. Given the importance of diagnosis to tailor treatment, there is a need to examine the prevalence and validity of SSD.

**Methods**: Adult patients attending the Michael G. DeGroote Pain Clinic referred for psychological services were assessed for SSD using a structured clinical interview, the Diagnostic Assessment Research Tool (DART). Patients also completed psychometric measures assessing several pain related constructs, including pain intensity, catastrophizing, disability, kinesiophobia, depression, anxiety, and somatic symptoms.

**Results & Discussion**: Two hundred and fifty-four patients with chronic pain were included in the study. Findings suggest that the majority of patients presenting for psychological services within a chronic pain clinic met criteria for SSD. Patients who were diagnosed with SSD had significantly higher scores on pain-related measures such as pain catastrophizing, kinesiophobia, and disability, and symptoms of anxiety, but not on pain intensity, somatic symptoms, and symptoms of depression. Risk factors such as being female and reporting a history of physical or sexual abuse were significant in the sample, where demographic factors such as unemployment, education, and age were not.

**Conclusion**: Findings suggest that DSM-5 SSD is highly prevalent in patients with chronic pain presenting for psychological services. Preliminary support for the validity of the SSD diagnosis was also provided, related to associations with kinesiophobia, catastrophizing, anxiety, and disability.

## Early Experience of Having a Full-time Pain Consultant Working for the UK Military. Does this Assist Earlier Rehabilitation and Treatment?

Jon Norman^a,*^

^a^Medical Division, Defence Medical Rehabilitation Centre, Stanford-on-Soar, Leicestershire, UK

**CONTACT** Jon Norman jonathan.norman114@mod.gov.uk

© 2020 The Author(s). Published with license by Taylor & Francis Group, LLC.

This is an Open Access article distributed under the terms of the Creative Commons Attribution License (http://creativecommons.org/licenses/by/4.0/), which permits unrestricted use, distribution, and reproduction in any medium, provided the original work is properly cited.

**Background**: The UK military has 155,000 personnel widely based in the UK and the world. A pain clinic at the national rehabilitation center (DMRC) was until 2016 not staffed bar 1 full time nurse with a weekly visiting consultant. DMRC is not a hospital and all services provided on site need to be risk assessed.

**Method**: Pain interactions were recorded for 2 years following the introduction of an onsite civilian pain consultant. Data includes treatments performed locally and requested beyond, waiting times and patient satisfaction.

**Results**: During this period the UK military was not involved in conflict. Despite this 20% of the UK was not judged fit to fight. 50% of cases were training injury, 30% sport, 15% mental health issues.

3083 patients attend DMRC in years 2016/7.74% reported pain (average BPI impact 6.25, severity 7), 90% had an anxiety or depression issue. The pain team assessed 768 and arranged intervention for 325 patients. New on-site services included epidurals, radiofrequency to facet or nerves and local neuromodulation reducing waiting times from 6 months (via the NHS) to 2 weeks and psychological assessment. Waiting times for assessment fell from 16 to 1 week. All patients assessed were satisfied or highly satisfied with their service and judged it useful. No significant complications were recorded.

**Conclusion**: The expansion of the on-site pain service improved speed of assessment and treatment. In future work we will see this improves the outcome of on-site rehabilitation as we increasingly involved earlier in the rehabilitation journey.

## Developing a Clinically Useful Short Form of the Tampa Scale of Kinesiophobia (TSK-11) in a Tertiary Care Chronic Pain Clinic

Etienne J. Bisson http://orcid.org/0000-0002-0649-3550^a,b,c,d,*^, Laura Katz^e^, Jennifer Bossio^a,f^, Tom Doulas^a^, Mary Anne Good^a^, Kyle Vader http://orcid.org/0000-0002-0667-9726^a,h^, Rosemary Wilson http://orcid.org/0000-0003-3262-243X^b,c^, Scott Duggan^a,c,i^

^a^Chronic Pain Clinic, Kingston Health Sciences Centre-Hotel Dieu Hospital Site, Kingston, Ontario, Canada; ^b^School of Nursing, Queen’s University, Kingston, Ontario, Canada; ^c^Department of Anesthesiology and Perioperative Medicine, Queen’s University, Kingston, Ontario, Canada; ^d^Centre for Neuroscience Studies, Queen’s University, Kingston, Ontario, Canada; ^e^Michael G. DeGroote Pain Clinic, McMaster University, Hamilton, Ontario, Canada; ^f^School of Medicine, Queen’s University, Kingston, Ontario, Canada; ^g^School of Rehabilitation Therapy, Queen’s University, Kingston, Ontario, Canada; ^h^Department of Biomedical and Molecular Sciences, Queen’s University, Kingston, Ontario, Canada

**CONTACT** Etienne J. Bisson etienne.bisson@kingstonhsc.ca

© 2020 The Author(s). Published with license by Taylor & Francis Group, LLC.

This is an Open Access article distributed under the terms of the Creative Commons Attribution License (http://creativecommons.org/licenses/by/4.0/), which permits unrestricted use, distribution, and reproduction in any medium, provided the original work is properly cited.

**Introduction/aims**: The Tampa Scale of Kinesiophobia (TSK-11) is a common measure to assess pain-related fear among patients with chronic pain, in line with a biopsychosocial assessment framework. In order to reduce patient burden (time to complete), clinician burden (scoring time), and increase clinical utility, a shorter version of this measure is needed. Thus, we are seeking to develop and validate a short-form TSK with good psychometric properties with the end goal of encouraging uptake of this tool as a gold-standard across chronic pain centers and researchers.

**Methods**: The Kingston Health Sciences Center (KHSC) Chronic Pain Clinic (Kingston, ON) assesses biopsychosocial characteristics (including TSK-11) for all patients enrolled. Patient-reported baseline data from this registry was extracted from November 2017 to October 2019 (N = 933, M age = 53.5 ± 15.7; 63% female). Data analyses included: 1) factor analyses and subsequent item reduction of the TSK-11, 2) Cronbach’s alpha’s to evaluate the scale’s internal consistency, and 3) correlations between the new short-form TSK and measures reflecting constructs of the fear-avoidance model (e.g. pain catastrophizing, pain severity, depression) to evaluate the concurrent validity of the new measure.

**Results:** A 2-factor structure was confirmed from the TSK-11 and the final model after item reduction resulted in a 7-item TSK (TSK-7) with 61.2% explained variance and Cronbach’s alphas of 0.76 and 0.70 for each factor. High correlations were found between the TSK-7 and TSK-11 (r = 0.96), along with similar correlation between the TSK7 and TSK11 with pain severity (r = 0.33 vs. 0.34), pain catastrophizing (r = 0.58 vs. 0.57) and depression (r = 0.46 vs. 0.45).

**Discussion:** The TSK-7, a shorter form of the TSK-11, was shown to have good psychometric properties and concurrent validity. This shorter form could help to standardize and simplify questionnaires across chronic pain centers in order to facilitate patient care as well as cross-site research collaboration.

## Effectiveness of Self-Help Acceptance and Commitment Therapy in Comparison to an Education Intervention to Manage Chronic Pain

Marie-Eve Martel^a,*^, M. Gabrielle Pagé^b^ and Frédérick Dionne^a^

^a^Department of Psychology, Université Du Québec À Trois-Rivières (UQTR),Trois-Rivières, Québec, Canada; ^b^Centre De Recherche, Centre Hospitalier De l’Université De Montréal, & Department of Anesthesiology and Pain Medicine, Université De Montréal, Montreal, Québec, Canada

**CONTACT** Marie-Eve Martel Marie-Eve.Martel1@uqtr.ca

© 2020 The Author(s). Published with license by Taylor & Francis Group, LLC.

This is an Open Access article distributed under the terms of the Creative Commons Attribution License (http://creativecommons.org/licenses/by/4.0/), which permits unrestricted use, distribution, and reproduction in any medium, provided the original work is properly cited.

**Objective**: In recent years, increasing efforts have been made to improve accessibility to evidence-based psychological treatments for chronic pain, notably with “self-help” interventions. This study aimed to evaluate the effectiveness of guided self-help interventions (web-based and bibliotherapy) based on Acceptance and Commitment Therapy (ACT) in comparison to an education intervention among adults living with chronic pain.

**Methods**: Participants (N = 297) were randomly assigned to a web-based ACT intervention group, an ACT-based bibliotherapy group, or an active control group receiving education on pain through online brochures. Participants completed self-reported online questionnaires at baseline, after the 9-week intervention, and at three- and six-month follow-ups. The primary outcome was pain disability and secondary outcomes were depression, anxiety, and quality of life.

**Results**: Results of mixed linear models showed statistically significant interaction effects between time and groups for pain disability (*F* = 2.91, *p* =.009) and anxiety (*F* = 2.26, *p* =.037) in favor of the bibliotherapy group. Results also showed significant main effects for time in terms of depression (*F* = 13.18, *p* =.0001) and quality of life (*F* = 23.96, *p* =.0001) for all three interventions.

**Discussion/Conclusions**: Findings suggest all three self-help intervention formats can lead to reductions in pain disability, depression, anxiety, and improvements in quality of life but a guided self-help intervention administered through ACT bibliotherapy may lead to greater reductions in pain disability and anxiety compared to web-based ACT interventions or an education intervention on pain.

## Randomized Comparison of Hands-on versus Video-based Training Program Designed to Enhance Pelvic Floor Examination in Patients Presenting with Chronic Pelvic Pain

Maria Giroux http://orcid.org/0000-0002-3401-262X^a,*^, Suzanne Funk^b^, Erwin Karreman^c^, Huse Kamencic^a^, Rashmi Bhargava^a^

^a^Obstetrics & Gynecology, University of Saskatchewan, Regina, Saskatchewan, Canada; ^b^Pelvic Floor Physiotherapy, Emerald Physiotherapy and Rehabilitation, Regina, Saskatchewan, Canada; ^c^Statistics, Saskatchewan Health Authority, Regina, Saskatchewan, Canada

**CONTACT** Maria Giroux mag640@mail.usask.ca

© 2020 The Author(s). Published with license by Taylor & Francis Group, LLC.

This is an Open Access article distributed under the terms of the Creative Commons Attribution License (http://creativecommons.org/licenses/by/4.0/), which permits unrestricted use, distribution, and reproduction in any medium, provided the original work is properly cited.

**Objective**: The purpose of this study was to compare the effectiveness of hands-on vs video-based training of a comprehensive assessment of the pelvic floor musculature on a pelvic model.

**Methods**: A randomized single-blinded trial was conducted between January 16 and November 19, 2018. 46 participants were enrolled and randomized to video (n = 23) and hands-on (n = 23) groups. Both groups underwent pre- training assessment that consisted of a written examination and an Objective Structured Clinical Examination (OSCE). Both groups had a didactic session. The video group then viewed instructional video and the hands-on group underwent hands-on training session with a pelvic floor physiotherapist. Both groups then underwent a post-training assessment. Primary outcome measure was the change in assessment scores from pre- to post-training. Secondary outcome measure was usefulness of the training program for clinical practice.

**Results**: The mean written assessment and OSCE scores improved significantly pre- and post-training in both hands- on and video-based training groups (*p* < .001). There was no statistically significant difference in the degree of improvement of the mean written assessment scores (*p* = .19), OSCE scores (*p* = .10), and comfort level (*p* = .19) between groups. The training program was useful for clinical practice.

**Conclusions**: Both video and hands-on are effective training methods. There is no difference in degree of improvement of assessment scores between both methods. This study presents a new effective multidisciplinary training program for teaching the assessment of the pelvic floor musculature to identify a possible muscular cause or contribution to chronic pelvic pain and provide early referral for appropriate treatment.

## L1 DRG Pulsed Radiofrequency Treatment Is a Therapeutic Option for Chronic Orchialgia

Rahul Pathak^a,*^ and Aaron Kirschner^b^

^a^Allevio Pain Clinic, Wasser Pain Management Centre, University of Toronto, Toronto, Ontario, Canada; ^b^Division of Neurology, McMaster University, Hamilton, Ontario, Canada

**CONTACT** Rahul Pathak Rahul.Pathak@allevioclinic.com

© 2020 The Author(s). Published with license by Taylor & Francis Group, LLC.

This is an Open Access article distributed under the terms of the Creative Commons Attribution License (http://creativecommons.org/licenses/by/4.0/), which permits unrestricted use, distribution, and reproduction in any medium, provided the original work is properly cited.

**Introduction:** Chronic orchialgia (testicular pain), defined as constant testicular pain for ≥3 months that significantly interferes with daily activities, is common, occurring in approximately 2.5% of all urology office visits. Treatment can often be difficult, owing to a lack of standard treatment protocols.

**Methods:** We present the case of a 38-year-old man with chronic left orchialgia following a ruptured varicocele for 2 years rated between 6/10 and 8/10 on a visual pain scale. Ultrasound-guided ilioinguinal block was trialed, which provided excellent relief for 2 months. This was followed by ilioinguinal nerve pulsed radiofrequency which provided longer-term but incomplete pain relief.

**Results:** Left L1 dorsal root ganglion (DRG) pulsed radiofrequency treatment resulted in 95% pain relief. The patient was contacted 11 months after the procedure and remained pain-free.

**Discussion/Conclusion:** We conclude that L1 DRG pulsed radiofrequency treatment is a therapeutic option for chronic orchialgia.

## The Alberta Pain Strategy – A Truly Provincial Collaboration

John X. Pereira^a,*^, Tracy Wasylak^b^, Susan Sobey-Fawcett^c^, Lori Montgomery http://orcid.org/0000-0001-6338-6455^d^, Nivez Rasic^e^ and Robert Tanguay^f^

^a^Family Medicine, University of Calgary, Calgary, Alberta, Canada; ^b^Alberta Health Services, Strategic Clinical Networks, Nursing, University of Calgary, Calgary, Alberta, Canada; ^c^Strategic Clinical Networks, Alberta Health Services, Calgary, Alberta, Canada; ^d^Family Medicine & Anesthesiology Perioperative and Pain Medicine, University of Calgary, Calgary, Alberta, Canada; ^e^Anesthesia, University of Calgary, Calgary, Alberta, Canada; ^f^Psychiatry & Surgery, University of Calgary, Calgary, Alberta, Canada

**CONTACT** John X. Pereira john@johncpc.com

© 2020 The Author(s). Published with license by Taylor & Francis Group, LLC.

This is an Open Access article distributed under the terms of the Creative Commons Attribution License (http://creativecommons.org/licenses/by/4.0/), which permits unrestricted use, distribution, and reproduction in any medium, provided the original work is properly cited.

A multi-stakeholder group of over 360 individuals, led by Alberta Health Service’s Strategic Clinical Networks™ and the Pain Society of Alberta, have come together to create The Alberta Pain Strategy and outline a coordinated approach to managing pain across the lifespan and across the province. The Strategy pays special attention to acute pain, transitional pain, chronic pain, cancer-related pain, and pain related to a palliative illness, condition, or disease. It is built on the principles of Quadruple Aim to improve patients’ and families’ experiences; patient and population health outcomes; the experience and safety of our people; and financial health and value for money. The Strategy’s six guiding principles are as follows: a culture of quality, the patient and family experience, prevention, health care equity, engagement and collaboration, and evidence-informed practice. Three focus areas of acute pain, chronic pain and opioid use in pain management were selected, with pain research and education being priorities that span all domains. Within each focus area, working groups identified priorities that would advance The Strategy’s overarching principles and objectives. The Alberta Pain Strategy was released in October 2019 and is available at painab.ca.

## I’ve Got the Power: People in Pain and Their Journey through Coaching

Mark Nazemi http://orcid.org/0000-0003-3495-6273^a,*^, Dorota Hedzelek^a^, Nicole Kern^b^, Alyson Kerr^b^, Mikayla Mazur^b^, Jen Hanson^a^, Maria Hudspith^a^

^a^Pain BC, Vancouver, British Columbia, Canada; ^b^UBC, School of Social Work, Vancouver, British Columbia, Canada

**CONTACT** Mark Nazemi mark@painbc.ca

© 2020 Pain BC Society. Published with license by Taylor & Francis Group, LLC.

This is an Open Access article distributed under the terms of the Creative Commons Attribution License (http://creativecommons.org/licenses/by/4.0/), which permits unrestricted use, distribution, and reproduction in any medium, provided the original work is properly cited.

Health and wellness coaching is an emerging intervention that may help an individual move towards living a more balanced life by improving their quality of life through learning self-managing strategies and adopting supportive behaviours. The purpose of this study is to review the effectiveness of Pain BC's Coaching for Health. The program offers free, 12 telephone coaching sessions aimed to help people living with pain learn self-management skills, regain function, make meaningful changes in their lives and improve their well-being. The program is accessed through a referral from a licensed health care provider, including primary care providers, physiotherapists, clinical counsellors, and more. A qualitative approach was used to analyze 128 client files out of 150 clients. An open coding approach was used to organize the raw data and look for common themes and trends across sessions. The results highlighted the effectiveness of the Coaching for Health program by providing an opportunity for clients to improve their quality of life, keep them accountable with pain management goals, improve confidence, and give them the autonomy to move towards a balanced life. Future improvements to the program include integrating a trauma-sensitive and mindfulness-based approach.

## Breaking Barriers to Care: Learnings from a Pain Support Line

Mark Nazemi http://orcid.org/0000-0003-3495-6273^a,*^, Dorota Hedzelek^a^, Jen Hanson^a^, Maria Hudspith^a^

Pain BC Society , Vancouver, British Columbia, Canada

**CONTACT** Mark Nazemi mark@painbc.ca

© 2020 Pain BC Society. Published with license by Taylor & Francis Group, LLC.

This is an Open Access article distributed under the terms of the Creative Commons Attribution License (http://creativecommons.org/licenses/by/4.0/), which permits unrestricted use, distribution, and reproduction in any medium, provided the original work is properly cited.

**Introduction/Aim:** In a recent Canada wide poll, one in three Canadians (34%) reported experiencing pain that has lasted longer than three months. Thecomplexities of pain impact many aspects of a person's life including employment, personal relationships, sleep, mood, physical, and behavioural health. For many facing such challenges, access to social services and support whether in urban or rural regions, are often difficult and impacts recovery.

**Methods:** In this study, we examine longitudinal data collected from Pain BC’s Pain Support Line to identify the social determinants of health needs of people living with pain in British Columbia (BC), Canada. This free telephone-and-email based pain support service is unique in Canada offering a safe space for people to talk about pain and its impact on their life. A quantitative analysis was conducted based on caller data collected from 1,652 clients who used Pain BC’s free volunteer-run phone and email Pain Support Line service over a span of four years. The service also provides information on self-management tools, connections to community resources, help navigating the health system, and information about Pain BC’s other services and resources. 

**Discussion/Conclusions:** The Pain Support Line also aims to attend to each caller using empowering language applying compassionate, trauma-informed, strength-based, and person-centred approach. The data from the support line provides a snapshot of the specific determinants. In addition, the study illustrates the need for individualized support for people in pain by creating a low barrier service that provides social support, concrete resources, and connects people with appropriate local community-based health services.

## Creative Art as a Therapeutic Intervention During a Mindfulness Course in a Community-based Interdisciplinary Publicly Funded Pain Program

Louisa Mailis^a,*^, Alex Mailis^b^ and Angela Mailis http://orcid.org/0000-0001-5662-8989^c^

^a^Mindfulness Teacher & Certified Professional Coach, Pain and Wellness Centre, Vaughan, Ontario, Canada; ^b^Chiropractor & Professional Coach, Pain and Wellness Centre, Vaughan Ontario, Canada; ^c^Physical Medicine & Rehabilitation, University of Toronto, Vaughan Ontario, Canada

**CONTACT** Louisa Mailis louisa@thepwc.ca

© 2020 The Author(s). Published with license by Taylor & Francis Group, LLC.

This is an Open Access article distributed under the terms of the Creative Commons Attribution License (http://creativecommons.org/licenses/by/4.0/), which permits unrestricted use, distribution, and reproduction in any medium, provided the original work is properly cited.

**Background**: Participants in a Mindfulness course (for Stress and Pain Management) are asked to create an Art project that expresses their pain, their experiences and/or journey through the Mindfulness program, or any other insights that they have discovered along the way. They are given one week to complete the assignment and then asked to present it to the class.

**Aim**: The primary objective of this study is to describe the thematic nature of Artwork produced by chronic pain participants, as expression of their feelings and experiences.

**Methods**: All Artwork was viewed independently by 3 reviewers, each of whom marked the work on the basis of research questions. A qualitative approach using thematic analysis was undertaken. Disagreements between the reviewers were solved by collaborative discussion reaching consensus.

**Results**: Participants included 13 females, one male; mean age 43.6 years; age range 26–64 years; 9 were employed (6 full time, 3 part time), 3 were on disability and one retired. In regard to the Artwork, 5 major themes emerged as follows: 10/14 depicted a journey/transition; 13/14 showed expression of emotions (9/14 positive and negative emotions; 4/14 only positive emotions); 8/14 included nature themes; 6/14 used words to express their messages (3/14 combined nature and words while 3/14 used words only); and 5/14 had abstract themes. Examples of Art work and themes will be shown.

**Conclusions**: Artwork by chronic pan patients is a powerful tool for expressing their experiences and emotions even in the eyes of independent observers.

## The Chronic Pain Network: Evolution and Impact

Norm Buckley^a,*^, Kim Begley^b^ and Megan Groves^b^

^a^Anesthesiology, McMaster University, Hamilton, Ontario, Canada; ^b^Chronic Pain Network, Hamilton, Ontario, Canada

**CONTACT** Norm Buckley buckleyn@mcmaster.ca

© 2020 The Author(s). Published with license by Taylor & Francis Group, LLC.

This is an Open Access article distributed under the terms of the Creative Commons Attribution License (http://creativecommons.org/licenses/by/4.0/), which permits unrestricted use, distribution, and reproduction in any medium, provided the original work is properly cited.

A 2019 Angus Reid Institute poll, in partnership with PainBC, indicated that 1 in 5 Canadians’ quality of life and ability to function is limited by pain. 37% of those surveyed indicated that their access to pain medication has been limited, rendering them unable to obtain adequate relief. Traditionally, the role of patients in research was merely as subjects. However, recent years have seen an increase in emphasis on the value of input from those with lived experience – from helping to direct research to shaping policy.

With more than 20 research projects nearing completion, the Chronic Pain Network’s fifth year of funding through the Strategy for Patient Oriented Research has seen increased opportunities for those living with pain to engage with researchers, fostering dialogue and collaboration.

Network product PainPLUS CPN incorporates those with lived experience in selecting and rating articles, and producing lay summaries. The Clinical Research Network connects pain clinics across the country to run trials in a clinical setting, with a minimal dataset for a pain registry now being piloted. The Network’s Patient Engagement committee has created a document to guide researchers in the acknowledgment of contributions from those with lived experience when writing research papers, and a Patient Advisory Group allows researchers to seek feedback from those with lived experience. The Indigenous Research Advisory committee has compiled a compendium of resources for approaching Indigenous communities and is hosting workshops based on results from a recent survey. New ideas continue to take shape, growing as the Network matures.

## Exploring Future Physical Therapists’ Professional Identity in Regard to Pain

Zoe Leyland^a,*^, Dave Walton^b^ and Elizabeth Anne Kinsella^c^

^a^Health Rehabilitation Sciences, Western University, London, Ontario, Canada; ^b^School of Physical Therapy, Western University, London, Ontario, Canada; ^c^School of Occupational Therapy, Western University, London, Ontario, Canada

**CONTACT** Zoe Leyland zletwin@uwo.ca

© 2020 The Author(s). Published with license by Taylor & Francis Group, LLC.

This is an Open Access article distributed under the terms of the Creative Commons Attribution License (http://creativecommons.org/licenses/by/4.0/), which permits unrestricted use, distribution, and reproduction in any medium, provided the original work is properly cited.

**Background:** While pain is often the most common complaint when patients visit their clinicians, most clinicians are uncomfortable with addressing the issue of pain, and it has become increasingly recognized that current pain education for healthcare providers is inadequate. This is evidenced by current public health crises such as, the epidemic for chronic pain and the ‘opioid crisis.’ Pain education for health care professionals has traditionally been suboptimal. Through the Master of Physical Therapy program at Western University, an elective course, Understanding Pain in Rehabilitation (PT9551b) has been available for physical therapy trainees. The primary outcome for the course was a reflective journal. The true goal of the course; however, is that some students undergo a truly transformative change in the ways they see themselves as ‘providers of pain management’ or ‘providers of physical therapy for people in pain’, but it was not previously rigorously explored.

**Aim:** The primary outcome of this study was to explore physical therapy students lived experiences of the course and determining if the course shaped their professional identities as ‘pain management providers.’

**Methods:** Semi-structured interviews took place and the study followed an interpretive phenomenological approach. In following a phenomenological methodology, a small sample size of 8 participants were included.

**Results:** In conducting this project, it contributed to a larger project of developing a master’s level interprofessional pain management program for practicing clinicians that launched September 2019.

## Utilization of Mental Health Resources: Lessons Learned Using the Pediatric Pain Screening Tool to Triage Chronic Pain and Headache Patients

Monica L. Gremillion^a,*^, Nina Linneman^b^, Kim Anderson Khan^c^, Chasity Brimeyer^c^, Steven J. Weisman^c^ and Keri R. Hainsworth^c^

^a^Psychiatry, Children’s Hospital of Wisconsin, Milwaukee, Wisconsin, USA; ^b^Counseling Psychology, University of Wisconsin—Milwaukee, Milwaukee, Wisconsin, USA; ^c^Anesthesiology, Medical College of Wisconsin, Milwaukee, Wisconsin, USA

**CONTACT** Monica L. Gremillion mgremillion@chw.org

© 2020 The Author(s). Published with license by Taylor & Francis Group, LLC.

This is an Open Access article distributed under the terms of the Creative Commons Attribution License (http://creativecommons.org/licenses/by/4.0/), which permits unrestricted use, distribution, and reproduction in any medium, provided the original work is properly cited.

The Pediatric Pain Screening Tool (PPST; Simons et al., 2015) is used in our clinic as part of a new initiative to improve access to care. The lack of mental health providers embedded in pediatric pain clinics can limit access to care for those most in need. The PPST can identify patients needing more intensive follow up care (i.e., psychotherapy), but the measure has not previously been used to triage patients *prior* to their initial evaluation. Our clinic triaged 1196 patients over 22 months. The need to manage this extraordinary clinic volume with limited resources (one full-time psychologist and two part-time psychologists) required a novel approach to prospectively triage initial evaluations. We use the PPST to stratify patients into: multidisciplinary or medical-provider-only evaluations. Headache patients scoring <3 on the PPST see only a medical provider. Headache patients scoring ≥3 are evaluated by a medical provider-psychologist team, based on previously established clinical cutoffs. All chronic pain patients are evaluated by a multidisciplinary team, which is consistent with their higher PPST scores when compared to headache patients (*p* <.001). From our experience: 1) the PPST appropriately triaged patients 86% of the time; 2) 28% of patients only needed education, advice, or medications, which can be provided by medical providers; 3) 72% of patients needed more intensive evaluation by psychologists. Further, we identified that some medical providers needed additional support and training for evaluation of psychosocial issues, which can be provided by interdisciplinary case conferences.

## Development of a Group-based Physical Activity Goal Setting Workshop in a Tertiary Care Chronic Pain Clinic

Kyle Vader^a,*^, Tom Doulas^a^, Lori Selkirk^a^, Kerry Oulton^a^ and Scott Duggan^a^

Chronic Pain Clinic, Kingston Health Sciences Centre, Kingston, Ontario, Canada

**CONTACT** Kyle Vader kyle.vader@kingstonhsc.ca

© 2020 The Author(s). Published with license by Taylor & Francis Group, LLC.

This is an Open Access article distributed under the terms of the Creative Commons Attribution License (http://creativecommons.org/licenses/by/4.0/), which permits unrestricted use, distribution, and reproduction in any medium, provided the original work is properly cited.

**Background:** Physical activity and exercise can decrease pain severity and improve physical function among adults with chronic pain. Despite the benefits, engaging in physical activity and exercise is a challenge for adults with chronic pain. Previous research has shown that adults with chronic pain want support, tailored advice, and improved access to physical activity and exercise programming.

**Purpose:** To improve access to physical activity programming in the Chronic Pain Clinic at Kingston Health Sciences Center (KHSC), a group-based physical activity goal setting work- shop was developed.

**Description of Program:** The Chronic Pain Clinic at KHSC is an academic, tertiary care clinic that includes an interdisciplinary team of health care providers with experience and expertise in chronic pain management. The group-based physical activity goal setting workshop is theoretically rooted in social cognitive theory, whereby behavior change is conceptualized as a reciprocal interaction between a person (e.g. adult with chronic pain), environment (e.g. group-based physical activity goal setting workshop), and behavior (e.g. engaging in physical activity). Workshop development took an evidence-based approach and was developed based on a review of the literature, clinician expertise, and patient values. The physical activity workshop is two-hours in length, facilitated by a physiotherapist and/or occupational therapist, and designed for ten patients. It includes a workbook with content on pain science, the role of physical activity for chronic pain management, reflective exercises, and goal setting.

**Next Steps:** The workshop is currently being piloted, whereby feedback will be sought from multiple stake-holders, including referring clinicians, clinicians who deliver the workshop, as well as patients.

## #PartneringForPain: Mobilizing Patient Priorities for Pediatric Chronic Pain into Action

Justina Marianayagam^a,*^, Carley Ouellette^b^, Esther Fleurimond^c^, Kimberly Nelson^d^, Julie Drury^e^, Garry Salisbury^f^, Christine Lamontagne^h^, Paula Forgeron http://orcid.org/0000-0002-4686-9698^i^, Fiona Campbell^j^, Jennifer Stinson^k^ and Kathryn A. Birnie^l^

^a^Faculty of Medicine, Northern Ontario School of Medicine, Thunder Bay, Ontario, Canada; ^b^School of Nursing, McMaster University, Hamilton, Ontario, Canada; ^c^Gatineau, Quebec, Canada; ^d^Windsor, Ontario, Canada; ^e^Ottawa, Ontario, Canada; ^f^Ministry of Health and Long Term Care, Kingston, Ontario, Canada; ^g^Department of Anesthesiology and Pain Medicine, Children’s Hospital of Eastern Ontario, University of Ottawa, Ottawa, Ontario, Canada; ^h^School of Nursing, University of Ottawa, Ottawa, Ontario, Canada; ^i^Department of Anesthesia & Pain Medicine, The Hospital for Sick Children, Toronto, Ontario, Canada; ^j^Child Health Evaluative Sciences, The Hospital for Sick Children, Toronto, Ontario, Canada; ^k^Department of Anesthesiology, Perioperative and Pain Medicine, University of Calgary, Calgary, Alberta, Canada

**CONTACT** Justina Marianayagam jmarianayagam@nosm.ca

© 2020 The Author(s). Published with license by Taylor & Francis Group, LLC.

This is an Open Access article distributed under the terms of the Creative Commons Attribution License (http://creativecommons.org/licenses/by/4.0/), which permits unrestricted use, distribution, and reproduction in any medium, provided the original work is properly cited.

**Background**: The #PartneringForPain team engaged hundreds of individuals with pediatric chronic pain, family members, and healthcare providers in a JLA Priority Setting Partnership to identify the Top 10 priorities for pediatric chronic pain. There is now an urgent need to ensure these priorities are acted on by relevant stakeholders.

**Initiative/Methods**: An hour-long interactive round table discussion of the Top 10 priorities took place with 14 multidisciplinary pediatric chronic pain clinicians, 4 hospital administrators, and 2 people with lived experience through the Ontario Chronic Pain Network in April 2019. The session was co-facilitated by a patient partner and researcher/clinician project lead.

**Evaluation/Results**: Participants largely agreed that the top priorities reflected their own priorities for pediatric chronic pain research and/or care in Ontario (89% agree or strongly agree), were interested to act on at least one of the priorities (83% agree or strongly agree), felt able and empowered to act on at least one of the priorities (79% agree or strongly agree), and thought the session would make a difference to the Ontario Chronic Pain Network (90% agree or strongly agree). All agreed or strongly agreed it is important to partner/engage with patients and families to address these priorities and felt better informed about what priorities Canadians with chronic pain, their families, and clinicians have about treating pediatric chronic pain. Participants also described how they would change their clinical practice, research, or professional activities because of the session.

**Conclusions**: Our activities showcase knowledge translation and action around patient- and family-identified priorities.

## Education as a Core Conservative Treatment for Hip and Knee Osteoarthritis Patients in an Alberta Hip and Knee Clinic

Kira Ellis^a^, Sofia Azeez^b,*^, Katelyn Reczek^b^ and Emily Brockman^b^

^a^Alberta Health Services Bone and Joint Health Strategic Clinical Network, Calgary, Alberta, Canada; ^b^Alberta Bone and Joint Health Institute, Calgary, Alberta, Canada

**CONTACT** Sofia Azeez sazeez@albertaboneandjoint.com

© 2020 The Author(s). Published with license by Taylor & Francis Group, LLC.

This is an Open Access article distributed under the terms of the Creative Commons Attribution License (http://creativecommons.org/licenses/by/4.0/), which permits unrestricted use, distribution, and reproduction in any medium, provided the original work is properly cited.

**Background:** Each year, over 10,000 osteoarthritis (OA) patients referred to Albertan Hip and Knee Central Intake clinics are determined to be conservative management candidates and there are limited instructions for next steps to support the patient’s OA pain management and functional abilities. Conservative care includes all treatment strategies for OA outside of surgery. These treatments and self-management strategies may be offered by multiple health care disciplines and are patient centred, using a shared decision-making approach to determine the most appropriate options for each individual.

**Purpose:** To determine the impact of conservative care education and tools designed to promote self-management strategies in patients living with OA in their hip or knee joints.

**Methods:** Three touch points were designed to integrate into existing clinic workflows along with an OA Self-Management Toolkit for patients. The touch points include:

(1)  An introductory OA education class delivered to all patients whose referral was accepted to a clinic, while they wait for their medical consult.

(2)  A second, individually tailored class after the medical consult, for those patients who were designated as conservative management candidates.

(3)  One year of telephone support from a Patient Navigator for conservative management candidates. This is an OA expert who can provide counsel, advise on evidence-informed treatment options or lifestyle changes, and direction to other disciplines when appropriate. Outcome measures and patient satisfaction surveys are delivered on a set schedule to patients by email. Outcome measures included self-rating surveys of OA status and readiness to change, Western Ontario McMaster University Osteoarthritis Index (WOMAC), EQ-5D-5L, and Patient Specific Functional Scale (PSFS).

The OA Self-Management Toolkit include three tools:

(1)  Report Card: A patient reflection tool to document their experience with conservative treatments to date and identify goals and barriers to determine a treatment plan.

(2)  Treatment Menu: A bingo card of conservative treatment options based on the 2019 Osteoarthritis Research Society International Guidelines for the Non-Surgical Management of Knee, Hip and Polyarticular Osteoarthritis.

(3)  Resource Inventory: A list of local community resources for OA conservative care.

The delivery of the program is being tested at the Chinook Bone and Joint Clinic in Lethbridge, Alberta.

**Results:** Interim results have been completed for the evaluation. Approximately 79% of patients (N=33) were diagnosed with OA in knee joints, and half the patients (N=10) described symptoms of early disease. The majority of patients, 86%, indicated a readiness to change current self-management practices. At the 6-month mark, the WOMAC and EQ-5D-5L scores have shown minimal change. The PSFS findings show a slight improvement of 0.30 in the patients’ functional ability, with half of patients indicating stair climbing and walking as priority goals. Patient feedback on the education classes acknowledged the following areas as beneficial to patients: learning about OA and how it affects the joints; learning about available treatment options to suit every lifestyle; debunking misconceptions about exercise as a treatment for OA; and group sessions promote inclusion and a community. Areas for improvement to education content and delivery include: the role of supplements and cannabis; earlier access to information prior to the class; and patient testimonials as positive examples. The majority, 97.0%, of patients who attended the first education class found it valuable and would recommend it to others (N=52). Similarly, 79.2% of patients felt better equipped to manage their OA (N=43) and 69.8% found some information to be repetitive (N=38). Although 92% of patients found the second education class valuable (N=12), patient recruitment has been low and recruitment strategies have been modified at 6-months into the evaluation to encourage participation.

**Conclusion:** The group education classes improve patients’ knowledge about their condition, self-management activities and access to available tools. The interim results revealed minimal changes in WOMAC and EQ-5D-5L scores, highlighting a need for more sensitive surveys to capture functional and quality of life changes for conservative patients. The PSFS survey encouraged goal setting and results showed improvements in the patients’ functional ability. An analysis of the Patient Navigator touchpoints, outcome tools collected, and focus group interviews with clinic staff and surgeons will be conducted once the evaluation period is complete in order to determine the full impact on patient care and understanding of conservative management of OA.

### Acknowledgement

This work was developed with the guidance of the Hip and Knee Osteoarthritis Conservative Working Group Tri-leads: Kelly Dinsmore, Dr. Ted Findlay and Dr. Geoff Schneider – with special acknowledgement to Sheila Kelly and Shannon Stephenson for their clinical input and support. The vision, implementation and evaluation of the program was achieved in collaboration with the Bone and Joint Health Strategic Clinical Network and the Alberta Bone and Joint Health Institute.

## Rhoda Reardon’s Environmental Scan- 12 Years Later- “If You Don’t Know Your History You are Doomed to Repeat It”

D Norman Buckley http://orcid.org/0000-0002-1031-6813^a,*^, Angela M. Carol^b^

^a^Department of Anesthesia, McMaster University, Hamilton, Ontario, Canada; ^b^College of Physicians and Surgeons of Ontario, Assistant Clinical Professor, Department of Family Medicine, McMaster University, Hamilton, Ontario, Canada

**CONTACT** D Norman Buckley buckleyn@mcmaster.ca

© 2020 The Author(s). Published with license by Taylor & Francis Group, LLC.

This is an Open Access article distributed under the terms of the Creative Commons Attribution License (http://creativecommons.org/licenses/by/4.0/), which permits unrestricted use, distribution, and reproduction in any medium, provided the original work is properly cited.

This poster describes changes in the practice and regulatory environment concerning pain care in Canada from 2007 to the present. To support the development of the 2010 Canadian Opioid Guideline by the Federation of Medical Regulatory Authorities of Canada, Rhoda Reardon, a researcher with the College of Physicians and Surgeons of Ontario, carried out an environmental scan. It identified 10 items which family practitioners and patients thought would help to address the challenges of pain management. These challenges were believed to underlie prescribing practices around opioid use. Some of these have been realized at either a local, provincial or national level- amongst these are the creation of national guidelines for opioid prescribing, and ongoing updating of these guidelines; creation of a recognized medical specialty in pain management; access to mentors with expertise in pain and addiction for primary care practitioners; improvement of undergraduate curricula for medical trainees; access to prescription monitoring systems; continuing education programs for pain, addiction and prescribing; patient support groups. Some have not- fee codes recognizing the complexity of pain care; access to networks of clinics for interdisciplinary care, practical guidance for emergency departments and walk in clinics on how to manage chronic pain. The poster reviews the environmental scan, and presents a summary of items which have and have not been achieved. With a national pain task force underway and provincial pain strategies being developed, the examination of this environmental scan allows recognition of significant progress for pain care over the past 12 years.

## myoActivation, A Structured Therapeutic System for Assessment and Management of Chronic Pain: A Pediatric Case Series

Gillian Lauder^a,*^ and Nicholas West^a^

Anesthesiology Pharmacology & Therapeutics, University of British Columbia, Vancouver, British Columbia, Canada

**CONTACT** Gillian Lauder glauder@cw.bc.ca

© 2020 The Author(s). Published with license by Taylor & Francis Group, LLC.

This is an Open Access article distributed under the terms of the Creative Commons Attribution License (http://creativecommons.org/licenses/by/4.0/), which permits unrestricted use, distribution, and reproduction in any medium, provided the original work is properly cited.

**Introduction:** Chronic pain in children is a significant cause of distress and reduced quality of life.^1,2^ A large proportion of children have unrecognized myofascial components to their chronic pain presentation. myoActivation® is a novel, structured assessment and non-pharmacological therapeutic approach to both diagnosis and treatment of chronic myofascial pain.^3^

**Methods:** A detailed history of the timeline of lifetime trauma combined with a quick, structured and reproducible set of posture and movement tests help to easily identify myofascial components of chronic pain. The therapeutic component is delivered utilizing a needling technique, or myofascial release, focused to active myofascial trigger points, fascia in tension and tethered scars.^4^ Repeat, or catenated, cycles of intervention followed by repeated movement tests help unravel multiple sources of myofascial chronic pain. myoActivation is a low cost, simple interventional technique that can be included in the biopsychosocial approach to chronic pain management, which is effective in adults as well as children and adolescents.^5^

**Results:** myoActivation has been a treatment option as a component of multidisciplinary treatment in the BC Children’s Hospital Complex Pain Service for the last 3 years. The myoActivation process and has been used to assess and manage chronic myofascial pain in children ranging from 4 to 18 years, including non-verbal patients. We will present a review of the last 100 discharged pediatric cases to illustrate key features of this treatment program, outline discharge outcomes and make provisional recommendations for its wider implementation in this population.

**Conclusion:** The information from this retrospective project will be used to power a future prospective longitudinal research project investigating myoActivation as a component of multidisciplinary care in children and adolescents with chronic pain.

References1.Perquin, et al.
Pain. 2000
7;87(1):51–8.1086304510.1016/S0304-3959(00)00269-42.King, et al.
Pain. 2011;152(12):2729–38.2207806410.1016/j.pain.2011.07.0163.Lauder, et al. doi: 10.5772/intechopen.84377.4.Boyles, et al.
J Man Manip Ther. 2015;23(5):276–93.2695525710.1179/2042618615Y.0000000014PMC47683805.Lauder, et al.
Curr Trends Med; 1(1): 1–16.

## Psychometric Properties of the Evaluation of Program Benefit Scales (EPB)

Eleni G. Hapidou^a,*^ and Laura Katz^b^

^a^Michael G. DeGroote Pain Clinic, Hamilton Health Sciences and McMaster University, Hamilton, Ontario, Canada; ^b^Medical Centre, Michael G. DeGroote Pain Clinic, Hamilton Health Sciences and McMaster University Hamilton, Hamilton, Ontario, Canada

**CONTACT** Eleni G. Hapidou hapidou@hhsc.ca

© 2020 The Author(s). Published with license by Taylor & Francis Group, LLC.

This is an Open Access article distributed under the terms of the Creative Commons Attribution License (http://creativecommons.org/licenses/by/4.0/), which permits unrestricted use, distribution, and reproduction in any medium, provided the original work is properly cited.

**Aim**: To establish psychometric properties of a new measure on evaluating interdisciplinary chronic pain management program benefit in physical, emotional/mental and social domains.

**Methods**: Admission and discharge data from self-report questionnaires were obtained from 325 patients from a funded program and 325 patients from a publically funded program. Measures included the Evaluation of Program Benefit (EPB), Pain Program Satisfaction Questionnaire (PPSQ), Self-Evaluation Scale (SES), Tampa Scale of Kinesiophobia (TSK), Pain Disability Index (PDI), and Pain Catastrophizing Scale (PCS). Internal consistency and predictive validity of the EPB were demonstrated using Cronbach’s alpha and linear regressions.

**Results**:Cronbach’s alpha for the three scales of the EPB was 0.82. The physical domain of the EBP was significantly predicted (F = 22.78, *p* < .01) by the PDI (β = −0.26, *p* < .01), but not the TSK or PCS. The mental domain was significantly predicted (F = 19.87, *p* < .01) by the PDI (β = −0.23, *p* < .01) and the PCS (β = −0.18), *p* < .01), but not the TSK. Lastly, the social domain was significantly predicted (F = 5.40, *p* < .01) by the PDI (β = −0.13, *p* = .02), but not the TSK or PCS.

**Discussion**: The EBP demonstrates excellent internal consistency and criterion validity with the PDI being the most consistent predictor. Even though data are sourced from two distinct program groups with differing strength of change from admission to discharge, the measure continues to be highly internally consistent and valid and can be used to assess chronic pain program benefit overall.

## Use of the Central Sensitization Inventory in Females with Chronic Pelvic Pain

Adria Fransson, BHScPT^a,*^ and Laura Katz, PhD., C.Psych^a^

Michael G. DeGroote Pain Clinic, McMaster University Medical Centre, Hamilton, Ontario, Canada

**CONTACT** Adria Fransson fransson@hhsc.ca

© 2020 The Author(s). Published with license by Taylor & Francis Group, LLC.

This is an Open Access article distributed under the terms of the Creative Commons Attribution License (http://creativecommons.org/licenses/by/4.0/), which permits unrestricted use, distribution, and reproduction in any medium, provided the original work is properly cited.

**Introduction/Aim:** Central sensitization (CS) is a physiological response of the central nervous system demonstrated by hypersensitivity to both noxious and non-noxious stimuli. The Central Sensitization Inventory (CSI) identifies key symptoms associated with CS syndromes, however, minimal research has evaluated CS in patients with chronic pelvic pain (CPP). The aim of this study was to evaluate the CSI with psychometric measures completed by female patients enrolled in an 8-session interdisciplinary CPP group program offered at the Michael G. DeGroote Pain Clinic.

**Methods:** Patients completed questionnaires for the CPP program including the CSI and pain-related variables such as pain self-efficacy (PSEQ), fear of movement (TSK), quality of life (PPIQ), pain catastrophizing (PCS) and depression, anxiety and stress (DASS-21). Data were analyzed using descriptive statistics, and a linear regression was utilized to evaluate pain-related predictors of the CSI.

**Results:** Fifty-one females completed the assessment. The regression was significant (*F* = 9.74, *p* < .01) with pain catastrophizing (*β *= 0.49, *p* < .01), quality of life (*β *= −0.42, *p = *.02), depression (*β *= 0.47, *p* = .01), and anxiety (*β *= 0.38, *p* = .02) as significant predictors of the CSI, while fear of pain/re-injury (*β *= −0.09, *p* = .53), pain self-efficacy (*β *= −0.15, *p* = .32) and stress (*β *= −0.05, *p* = .71) were not.

**Discussion/Conclusion:** Results demonstrate that the CSI was significantly predicted by a number of pain-related measures in women attending an interdisciplinary CPP group program. This suggests that CPP is not just a “tissue issue” but involves the sensitization of the nervous system including cognitions, quality of life, and mental wellbeing. As such, CPP is a complex condition that requires an interdisciplinary approach to treatment.

## Practical Acute Pain Management for Patients Stabilized on Buprenorphine in Tertiary Care

Patrycja Vaid http://orcid.org/0000-0002-2728-1661^a^, Tricia Metz^b^, Nathaniel Morin http://orcid.org/0000-0002-9220-1648^c^, Jennifer Joo http://orcid.org/0000-0002-2552-3268^d^, Kayla Denness^a^, S. Monty Ghosh^b^, Jeremy Hamming^d^, Humeira Dhanji^a^, Roberta DeJong^a^, and Robert L. Tanguay^e^

^a^Acute Pain Service, Alberta Health Services, Calgary, Alberta, Canada; ^b^Alberta Health Services, Opioid Dependency Program, Calgary, Alberta, Canada; ^c^Alberta Health Services, Pharmacy Services, Calgary, Alberta, Canada; ^d^Alberta Health Services, Anesthesia, Calgary, Alberta, Canada; ^e^University of Calgary, Addictions Services, Calgary, Alberta, Canada

**CONTACT** Patrycja Vaid patrycja.vaid@ahs.ca Acute Pain Service, Alberta Health Services, Calgary, Alberta, Canada

© 2020 Alberta Health Services. Published with license by Taylor & Francis Group, LLC.

This is an Open Access article distributed under the terms of the Creative Commons Attribution License (http://creativecommons.org/licenses/by/4.0/), which permits unrestricted use, distribution, and reproduction in any medium, provided the original work is properly cited.

**Introduction**: Buprenorphine is a partial μ agonist gaining popularity in Canada for the treatment of opioid use disorder and chronic pain. Its long half-life and high affinity to the μ receptor may theoretically prevent traditional opioids from working as intended during an acute pain episode. Scarce evidence about patients stabilized on buprenorphine in acute pain consists of case reports where opposing views on whether or not this therapy should be continued are presented. Practice guidelines for managing patients stabilized on buprenorphine experiencing acute pain can help practitioners make informed care decisions.

**Methods**: A cross-departmental multidisciplinary working group of experts analyzed available evidence and created tertiary care guidelines and treatment algorithm.

**Results**: A collaborative multidisciplinary and interdepartmental approach should be taken for an individualized approach to care. The most responsible outpatient prescriber should be contacted to coordinate care with the inpatient team. Usual pain management strategies need to be considered, including: multimodal analgesia, adjuvant therapy, regional anesthetic techniques, neuraxial techniques, ketamine infusions, lidocaine infusions, and non-pharmacological strategies. Buprenorphine may be continued, increased, decreased or paused. Considerations include: whether or not the painful admission is planned and unplanned, a patient’s addiction history, the patient’s pain history, the patient’s daily buprenorphine dose and reason for use, and the severity of expected acute pain.

**Conclusions**: Being aware of potential drug interactions between buprenorphine and traditional opioids and exploring recommended treatment options during painful admissions may lead to improved safety, better patient outcomes and provide basis for further study.

## Chronic Pain, Social Determinants of Health, and the Need for Intersectoral Action

Owen Williamson^a,*^

^a^Department of Epidemiology and Preventive Medicine, Monash University, Melbourne, Australia

**CONTACT** Owen Williamson owen.williamson@monash.edu

© 2020 The Author(s). Published with license by Taylor & Francis Group, LLC.

This is an Open Access article distributed under the terms of the Creative Commons Attribution License (http://creativecommons.org/licenses/by/4.0/), which permits unrestricted use, distribution, and reproduction in any medium, provided the original work is properly cited.

One in five Canadians live with chronic pain.

Chronic pain is more common among vulnerable populations, the young, the elderly, females, Indigenous Peoples, Veterans, people with mental health and substance abuse disorders. It particularly affects populations subjected to social inequities and discrimination.

Chronic pain should be a public health policy priority.

Chronic pain is a “wicked” problem, socially complex, multi-causal with many interdependencies, with no clear solution and beyond the responsibility of any one organization or government department.

Understanding and dealing with interactions between chronic pain and the social determinants of health involves considering factors beyond the domain of the health sector, such as education, income, social status, the physical environment and social support networks. Addressing these factors, may be more important than modifying existing health services in mitigating inequities in the management of chronic pain.

The World Health Organization (WHO) Commission on the Social Determinants of Health stated unequivocally that governments need to refocus their public health policies to ensure action by all sectors of government to address “the causes of the causes” in order to improve population health. This principle particularly applies when factors that cause and maintain chronic pain are outside the control of the health system.

Working across government sectors can be difficult, with “ownership”, funding, reporting arrangements, departmental or agency culture, and language presenting challenges.

The WHO Health in All Policies approach may facilitate intersectoral engagement and co-operation in the development of policy aimed at addressing the epidemic of chronic pain.

## Responding to the Opioid Crisis by Developing an Online Curriculum for Future Physicians on Pain Medicine and Substance Use Disorder

Lisa Graves^a,*^, Richard Van Wylick^b^, Nancy Dalgarno^c^, Fran Kirby^d^, Klodiana Kolomitro http://orcid.org/0000-0003-1250-8146^c^, Bryan MacLeod^e^ and David Flusk^f^

^a^Family and Community Medicine, Western Michigan University Homer Stryker M.D. School of Medicine, Kalamazoo, Michigan, USA; ^b^Pediatrics, Office of Professional Development & Educational Scholarship, Queen’s University, Kingston, Ontario, Canada; ^c^Faculty of Health Sciences, Office of Professional Development & Educational Scholarship, Queen’s University, Kingston, Ontario, Canada; ^d^The Association of Faculties of Medicine of Canada, Ottawa, Ontario, Canada; ^e^Clinical Science Division, Northern Ontario School of Medicine, Thunder Bay, Ontario, Canada; ^f^Discipline of Anesthesia, Memorial University of Newfoundland, St. John’s, Newfoundland and Labrador, Canada

**CONTACT** Lisa Graves lisa.graves@med.wmich.edu

© 2020 The Author(s). Published with license by Taylor & Francis Group, LLC.

This is an Open Access article distributed under the terms of the Creative Commons Attribution License (http://creativecommons.org/licenses/by/4.0/), which permits unrestricted use, distribution, and reproduction in any medium, provided the original work is properly cited.

**Background**: The Association of Faculties of Medicine of Canada undertook a substantive initiative in 2019, supported by Health Canada titled the “AFMC Response to the Opioid Crisis” to develop a national, integrated, online curriculum for undergraduate (UG) medical students in pain medicine and substance use disorder.

**Objective**: The purpose of this initiative is to close the educational gaps and empower the next generation of physicians with knowledge, skills, and resources needed to diagnose, treat and manage pain and substance use.

**Methods**:

(1)  Conducted two environmental scans to rigorously analyze existing curricula and high-impact practices.
(2)  Convened educational curriculum leaders to articulate competencies and learning objectives required by physicians.
(3)  Planned, using emerging technology and advanced instructional design, a comprehensive curriculum relevant to all Canadian medical schools.

**Results**: A curriculum structure has been informed by the environmental scans and undergraduate curriculum leaders. Competencies and content have been established within pedagogical frameworks mapped to a spiral curricular structure, and aligned with a national framework for medical education. This curriculum, its competencies and objectives will be presented.

**Conclusions**: An innovative national, bilingual, curriculum in pain and substance use disorder is a pivotal component of a national response to the opioid crisis. Our plan is to evaluate and extend the curriculum’s reach beyond undergraduate medical education, to postgraduate and continuing professional development by providing a competency-based, evidence-informed approach that aims to close the knowledge gaps and empower new and practicing physicians.

## Long Term Outcomes of Chronic Pain Patients Attending a Community-based Interdisciplinary Pain (IDP) Program Funded by the Ontario Ministry of Health and Long Term Care (MOHLTC)

Angela Mailis http://orcid.org/0000-0001-5662-8989^a,*^, Jordan Robinson^b^, Shah Fatima Lakha^b^

^a^Physical Medicine & Rehabilitation, University of Toronto, Vaughan, Ontario, Canada; ^b^Pain & Wellness Centre, Vaughan, Ontario, Canada

**CONTACT** Angela Mailis angela.mailis@uhn.ca

© 2020 The Author(s). Published with license by Taylor & Francis Group, LLC.

This is an Open Access article distributed under the terms of the Creative Commons Attribution License (http://creativecommons.org/licenses/by/4.0/), which permits unrestricted use, distribution, and reproduction in any medium, provided the original work is properly cited.

**Background**: While outcomes for interdisciplinary pain programs funded by third parties are reporting success rate of 14-60% long term, there is a dearth of outcome data from publicly funded IDPs.

**Aim**: To describe demographic characteristics and evaluate the changes in outcomes of patients’ self reported pain, and emotional/functional status, following treatment in a Canadian community-based publicly funded IDP employing a customized, patient centered and shared decision making model.

**Methods**: This retrospective study was conducted on 218 well selected chronic pain patients, with 158 completing a 3-4-month IDP during 2016–19. Data collected upon exit (N = 158), at 6 months (N = 66) and 12 months (N = 46) after program completion, was as follows: demographic information, pain characteristics, emotional/functional status obtained by validated instruments and Global Impression of Change (GIC). Means of pre-and post-program variables were compared to assess changes of each patient’s “journey”.

**Results**: Male/female ratio was: 1:2.1(*p* < .5); mean age 45 ± 16 years (18–83 yrs). Outcome data indicated statistically and clinically significant improvement from pre-treatment to post-treatment at 3 months (program exit) and 6 and 12-months post exit respectively, on all emotional/functional batteries. Examples of improvement are: BPI Pain interference score 40%, 47% and 53%; Anxiety 40%, 30% and 50%; Catastrophizing 46%, 46% and 64%; GIC (much/very much improved) 77%, 46% and 76%, respectively.

**Conclusion**: A publicly funded IDP using a customized patient-centered approach proved to be highly effective for chronic pain both in the short (3–4 months) and long term (6 and 12 months), with improvement ranging between 29-77% in different measures.

## The Development of a Step-wise Care Program Targeting Individual’s Stage of Readiness for Change

Veronica Wong^a,*^, Greg Tippin^a^ and Laura Katz^a^

Michael G. DeGroote Pain Clinic, McMaster University Medical Centre, Hamilton, Ontario, Canada

**CONTACT** Veronica Wong wongver@hhsc.ca

© 2020 The Author(s). Published with license by Taylor & Francis Group, LLC.

This is an Open Access article distributed under the terms of the Creative Commons Attribution License (http://creativecommons.org/licenses/by/4.0/), which permits unrestricted use, distribution, and reproduction in any medium, provided the original work is properly cited.

**Introduction**: Consistent with best practice guidelines, the Michael G. DeGroote (MGD) Pain Clinic offers an interdisciplinary and biopsychosocial program to manage chronic pain, with an emphasis on self-management. However, there are patients who drop out of the program, or attend the program with little reported improvement. Improved outcomes may be associated with matching the type of treatment provided to a patient’s readiness to engage in a self-management approach to chronic pain, as theorized by the transtheoretical model of behavior change. Specifically, patients in the pre-contemplative stage, where little action for self-management is being considered, may not be ready for an 8-day self-management program, thus resulting in lower completion rates and poorer clinical outcomes. The aim of this study is to describe the ongoing development and evaluation of a single-session interdisciplinary intervention (“Introduction to Pain Management”) targeting patients in the “pre-contemplative” stage with the goal to improve their readiness for change.

**Methods**: Patients identified as in the pre-contemplative stage based on the Pain Stages of Change Questionnaire were recommended to participate in the Introduction to Pain Management single-session group prior to enrollment in the 8-day interdisciplinary pain management program. The group intervention consists of a 60–90 minute Pain Neuroscience Education class with a physiotherapist followed by a 60–90 minute Motivational Interviewing session with a psychologist.

**Preliminary Results & Discussion**: Preliminary results will be presented, such as changes in readiness for change on the Pain Stages of Change Questionnaire, and insights will be shared on the development of this novel readiness intervention.

## Does Homework Completion Impact Outcomes in a Group-Based Mind-Body Intervention for Chronic Pain?

Pamela Holens^a,*^, Jeremiah Buhler^b^, Martine Southall^b^, Liana Rock^b^ and Catherine Desorcy-Nantel^b^

^a^Department of Clinical Health Psychology, University of Manitoba, Winnipeg, Manitoba, Canada; ^b^Department of Psychology, University of Manitoba, Winnipeg, Manitoba, Canada

**CONTACT** Pamela Holens pholens@deerlodge.mb.ca Department of Clinical Health Psychology, University of Manitoba, Winnipeg, Manitoba, Canada

© 2020 The Author(s). Published with license by Taylor & Francis Group, LLC.

This is an Open Access article distributed under the terms of the Creative Commons Attribution License (http://creativecommons.org/licenses/by/4.0/), which permits unrestricted use, distribution, and reproduction in any medium, provided the original work is properly cited.

**Introduction:** Chronic pain is a widespread concern in Canada and abroad. Pharmacological and surgical interventions commonly fail to sufficiently increase emotional and physical functioning while reducing pain levels. Various theoretical approaches have been adopted by clinicians treating patients with chronic pain (e.g., cognitive-behavioral, acceptance-based, meditation and mindfulness, hypnosis, and mind-body interventions). Preliminary evidence for a Mind-Body intervention for chronic pain used with a military and police population revealed significant reductions in both pain intensity and disability (Buhler et al., 2020). The aim of the present study was to examine the influence of homework completion on treatment outcomes for this intervention.

**Methods:** Seventeen military and police personnel (both active and retired) completed a 10-week Mind-Body intervention for chronic pain. Treatment was offered in groups of 6–8 individuals and utilized materials from the workbook “Unlearn Your Pain” (Schubiner & Betzold, 2019).

**Results:** A series of multiple regression analyses revealed that homework completion did not predict changes in pain disability or pain intensity from pre-to post-treatment. Furthermore, analyses showed that the type of homework completed (i.e., writing exercises, mindfulness exercises, brain-retraining exercises) was not predictive of treatment outcomes.

**Discussion:** Contrary to other findings regarding the influence of homework completion and treatment outcomes, the findings from this study suggest that positive treatment gains can be made irrespective of homework completion. Though these findings are preliminary in nature given the small sample size, the implications are important as many individuals present with limited motivation to complete out-of-session activities.

## A Mind-Body Approach to Chronic Pain Management for Military, RCMP, and Veterans

Pamela Holens^a,*^, Jeremiah Buhler^b^, Liana Rock^b^, Adair Libbrecht^b^, Michelle Paluszek^c^, Catherine Desorcy-Nantel^b^, Brent Joyal^b^ and Martine Southall^b^

^a^Department of Clinical Health Psychology, University of Manitoba, Winnipeg, Manitoba, Canada; ^b^Department of Psychology, University of Manitoba, Winnipeg, Manitoba, Canada; ^c^Department of Psychology, University of Regoma, Regina, Saskatchewan, Canada

**CONTACT** Pamela Holens pholens@deerlodge.mb.ca

© 2020 The Author(s). Published with license by Taylor & Francis Group, LLC.

This is an Open Access article distributed under the terms of the Creative Commons Attribution License (http://creativecommons.org/licenses/by/4.0/), which permits unrestricted use, distribution, and reproduction in any medium, provided the original work is properly cited.

**Introduction:** Chronic pain is a significant concern among military, RCMP, and veterans of these forces. A wide variety of methods for treating chronic pain have been used with this population, ranging from the purely physical to the purely psychological. The goal of this study was to determine the effectiveness of a 10-week mind-body chronic pain intervention for relieving pain and pain-related disability.

**Methods:** Participants were nineteen adult individuals with chronic pain who were patients at the Operational Stress Injury Clinic at Deer Lodge Center, a clinic that specializes in treating the mental health needs of military, RCMP, and veterans. In this quasi-experimental study, participants completed a variety of pain-related questionnaires at pre- and posttreatment. Treatment was offered in groups of 6–8 individuals and utilized materials from the workbook “Unlearn Your Pain” (Schubiner & Betzold, 2019).

**Results:** Analyses revealed that participants’ pain ratings were significantly decreased following the 10-week treatment. Pain-related disability was also improved, and kinesiophobia trended in the anticipated direction.

**Discussion:** This mind-body approach to chronic pain treatment shows significant potential for decreasing the experience of chronic pain among military, RCMP, and veterans. Future research using a randomized controlled trial format appears warranted.

## Case Report: Acute Pain Management of Patient Receiving Injectable Opioid Agonist Treatment (iOAT) following Total Knee Arthroplasty

Patrycja Vaid http://orcid.org/0000-0002-2728-1661^a,*^, Patty Wilson^b^, Gregory Abelseth^c^, Jacqueline Klemann^d^, S Monty Ghosh^e^, Cristina Zaganelli^f^

^a^Acute Pain Service (APS), Alberta Health Services (AHS), Calgary, Alberta, Canada; ^b^Injectable Opioid Agonist Therapy (iOAT), Addiction and Mental Health,Alberta Health Services (AHS), Calgary, Alberta, Canada; ^c^Section of Orthooedics, Department of Surgery, University of Calgary, Calgary, Alberta, Canada; ^d^Orthopedics,Alberta Health Services (AHS), Calgary, Alberta, Canada; ^e^Calgary ODP,Alberta Health Services (AHS), Calgary, Alberta, Canada; ^f^Calgary iOAT Program, Alberta Health Services (AHS), Calgary, Alberta, Canada

**CONTACT** Patrycja Vaid patrycja.vaid@ahs.ca

© 2020 Alberta Health Services. Published with license by Taylor & Francis Group, LLC.

This is an Open Access article distributed under the terms of the Creative Commons Attribution License (http://creativecommons.org/licenses/by/4.0/), which permits unrestricted use, distribution, and reproduction in any medium, provided the original work is properly cited.

**Introduction**: Patients requiring total knee arthroplasty (TKA) frequently suffer with chronic pain. Problematic opioid misuse ranges between 19-21% among those with chronic pain, and 8–12% struggle with Opioid Use Disorder (OUD) (Vowles KE, McEntee ML, Julnes PS, Frohe T, Ney JP, van der Goes, David N. Rates of opioid misuse, abuse, and addiction in chronic pain: A systematic review and data synthesis. Pain. 2015;156(4):569-76). One treatment for OUD is Opioid Replacement Therapy (ORT), which can be accomplished with injectable, or oral Opioid Agonist Treatment (iOAT, oOAT). Patients requiring iOAT represent a small fraction of individuals with OUD, and an even smaller fraction of individuals requiring TKR.

**Case Report**: We present a case report of a patient who required a TKR while receiving iOAT & oOAT of 4075 mg of oral daily morphine equivalent dose (MED) for OUD and chronic pain. Patients using medications for OUD present a clinical challenge during the perioperative period (Israel JS, Poore SO. The clinical conundrum of perioperative pain management in patients with opioid dependence: Lessons from two cases. Plast Reconstr Surg. 2013;131(4):658e; Macintyre PE, Russell RA, Usher K, Gaughwin M, Huxtable CA. Pain relief and opioid requirements in the first 24 hours after surgery in patients taking buprenorphine and methadone opioid substitution therapy. Anaesth Intensive Care. 2013;41(2):222). Effective acute pain management of patients on ORT is complicated by opioid tolerance, hyperalgesia, dependence and other psychosocial issues (Macintyre PE, Russell RA, Usher K, Gaughwin M, Huxtable CA. Pain relief and opioid requirements in the first 24 hours after surgery in patients taking buprenorphine and methadone opioid substitution therapy. Anaesth Intensive Care. 2013;41(2):222). Despite this challenge, the patient’s post-surgical pain was well managed using a multimodal and collaborative multidisciplinary approach. The coordinated care between the Acute Pain Service, Addictions Service, Anesthesia, Orthopedic Surgery, Pharmacy, Social Work, and Transition Services all contributed to this patient’s positive experience.

**Discussion**: Marginalized populations such as those using iOAT require the utmost care and compassion to ensure safety and promote positive outcomes post operatively. During the current opioid crisis, it is essential to become familiar with iOAT/oOAT, work collaboratively, draw on expert opinion and be prepared for the complex care required for treating the acute pain of patients on ORT in tertiary care.

## Case Studies of Clinical Pharmacogenomics Testing in Pain: Clinical, Practical and Ethical Considerations

Laura Murphy http://orcid.org/0000-0002-4787-8879^a,*^, Laura Flynn^b^, Daniel Z. Buchman http://orcid.org/0000-0001-8944-6647^c^, John Flannery^d^, Andrea D. Furlan http://orcid.org/0000-0001-6138-8510^d^

^a^Department of Pharmacy, University Health Network, Leslie Dan Faculty of Pharmacy, University of Toronto, Toronto, Ontario, Canada; ^b^Leslie Dan Faculty of Pharmacy, University of Toronto, Toronto, Ontario, Canada; ^c^Dalla Lana School of Public Health, Joint Centre for Bioethics, University Health Network, University of Toronto, Toronto, Ontario, Canada; ^d^Faculty of Medicine, University of Toronto, Toronto Rehab,University Health Network, Toronto, Ontario, Canada

**CONTACT** Laura Murphy laura.murphy@uhn.ca

© 2020 The Author(s). Published with license by Taylor & Francis Group, LLC.

This is an Open Access article distributed under the terms of the Creative Commons Attribution License (http://creativecommons.org/licenses/by/4.0/), which permits unrestricted use, distribution, and reproduction in any medium, provided the original work is properly cited.

Pharmacogenomic (PGx) testing has the potential to improve outcomes for patients with pain. PGx is the study of the role the genome plays in drug response, with a focus on genetic variants that affect pharmacokinetics and pharmacodynamics. It is an emerging field of medicine, with tests marketed directly to patients with pain and healthcare professionals. Approximately 60% of variation in individual responses to pain medications relate to genetic polymorphisms. Other medical specialties are more advanced in the implementation of PGx, for example cardiology and mental health. There are known gene-drug interactions with associated evidence-informed prescribing actions. Currently, there is very limited evidence supporting the use of PGx for patients living with pain and these tests are unfamiliar to many clinicians. We present three case studies of patients living with pain who pursued PGx testing to illustrate clinical, practical, and ethical considerations. Their PGx results provided insight into lack of response to therapies, risk of potential adverse effects, and potential future drug interactions. We discuss challenges with PGx report interpretation and usefulness. PGx testing raises ethical issues including those related to consent, privacy, confidentiality, potential secondary uses of genetic data, and return of results. These cases illustrate the need for further research and education to navigate integration of PGx in pain medicine.
